# Poster Session II A324 THE NAUSEA ATLAS: IDENTIFYING AND CHARACTERIZING NAUSEA SUBTYPES USING PATIENT-REPORTED INFORMATION

**DOI:** 10.1093/jcag/gwaf042.324

**Published:** 2026-02-13

**Authors:** S Sonaiya, K Y Hui

**Affiliations:** University of Nevada Las Vegas Kirk Kerkorian School of Medicine, Las Vegas, NV; Internal Medicine, Johns Hopkins University School of Medicine, Baltimore, MD

## Abstract

**Background:**

Across the practice of medicine, nausea is a common and poorly understood symptom. Although many pharmacologic and other interventions are available to treat nausea, there is little evidence to guide the prioritization of treatment options. Despite some knowledge of mechanistic pathways, such as dysautonomia and GI dysfunction, the delineation of clinical phenotypes in nausea also remains poor.

**Aims:**

We sought to comprehensively catalog and organize the experiences of patients with nausea, using self-reported data, for the dual purposes of A) guiding treatment based on improved history-taking and B) generating hypotheses for future studies of pathophysiologic pathways as targets for novel therapies.

**Methods:**

We performed an anonymous online survey of patients recently documented in the medical record as experiencing nausea, who answered over 200 multiple-choice questions than spanned demographics, quality of life, and multiple aspects of nausea: burden and severity, descriptive characteristics, causes, exacerbators, and effectiveness of medications and non-medication therapies. We enrolled 2,298 participants, many of whom experienced multiple types of nausea, yielding 3,669 complete descriptions of individual nausea experiences.

**Results:**

Respondents experienced a high burden of nausea, as they most commonly reported having daily nausea for over one year, with moderate impairment of ability to perform tasks. The most common exacerbator of nausea was “eating or drinking anything.” Among all respondents, ondansetron was the most effective anti-nausea treatment, followed by promethazine, lorazepam, marijuana, and metoclopramide (Fig 1). For most medications, route of administration (e.g. PO vs. IV) had a minimal impact on effectiveness, with the notable exception of metoclopramide being substantially more effective in IV form. The most effective non-medication treatment was marijuana, which was considerably more effective than dronabinol. In pairwise analysis between causes of nausea and effectiveness of medications, the top statistical association showed that domperidone is more effective in gastroparesis (FDR = 0.0004, Wilcoxon test). The top association for non-medication treatments identified that talk therapy is more effective for anxiety-associated nausea (FDR = 2.5 x 10^-31^, Wilcoxon test). Using principal components analysis, we highlighted certain causes of nausea associated with unique descriptor profiles, including IBS and GLP1 agonist medications.

**Conclusions:**

To our knowledge, this is the first large-scale and comprehensive characterization of patients with chronic, severe nausea. It provides a novel evidence basis for nausea treatment guided by clinical characteristics, and it highlights the potential to uncover basic knowledge about nausea mechanisms.

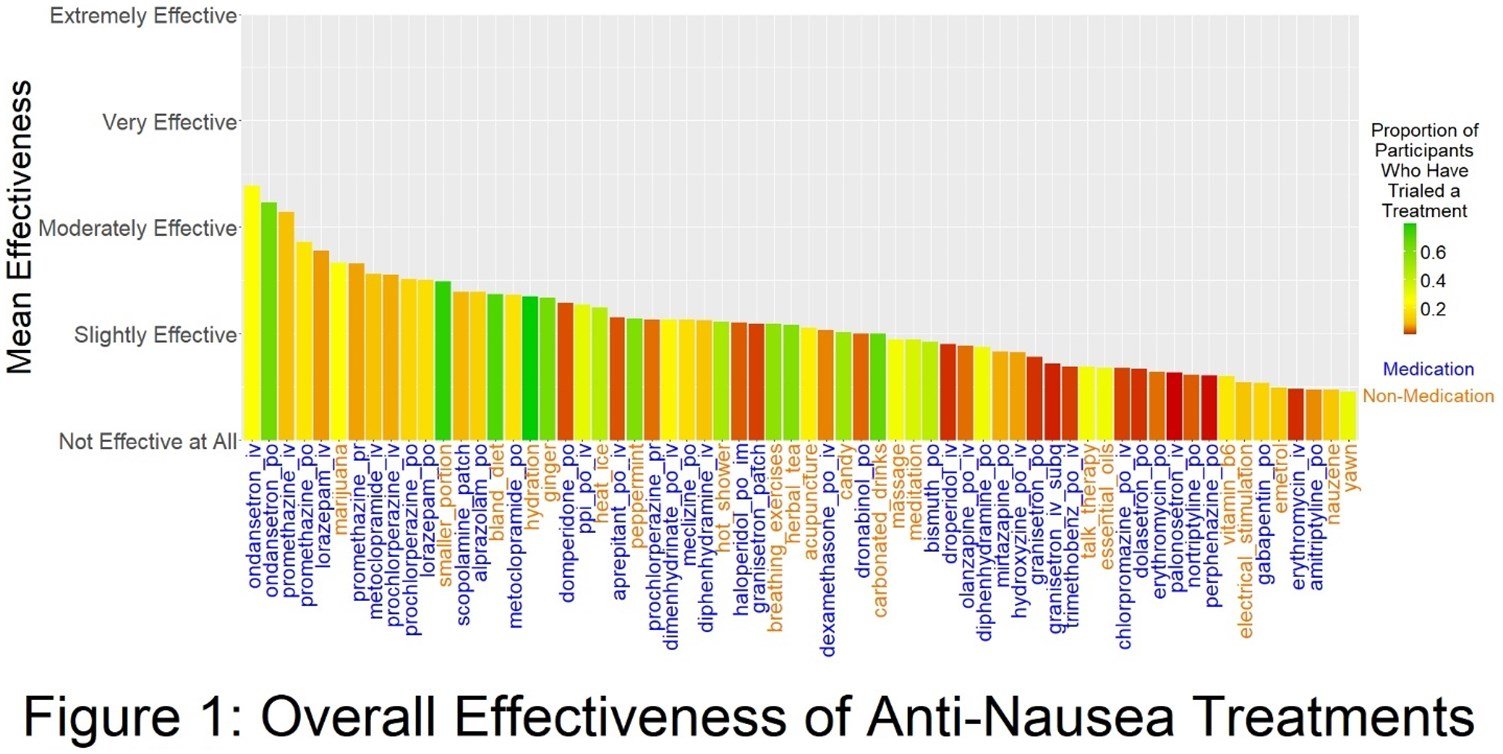

**Funding Agencies:**

None

